# Effect of prematurity on genome wide methylation in the placenta

**DOI:** 10.1186/s12881-019-0835-6

**Published:** 2019-06-28

**Authors:** Jessica Schuster, Alper Uzun, Joan Stablia, Christoph Schorl, Mari Mori, James F. Padbury

**Affiliations:** 1 Pediatrics, Women & Infants Hospital, Providence, Rhode Island 02905 USA; 2 Pediatrics, Center for Computational Molecular Biology, Brown Medical School, Brown University, Providence, Rhode Island 02906 USA; 3 Molecular Biology, Cell Biology and Biochemistry, Brown University, Providence, Rhode Island 02906 USA; 4 Pediatrics and Genetics, Hasbro Children’s Hospital, Providence, Rhode Island 02905 USA; 5 Providence, USA

**Keywords:** Preterm birth, Fetal programming, Epigenetics, Placenta

## Abstract

**Background:**

Preterm birth is a significant clinical problem and an enormous burden on society, affecting one in eight pregnant women and their newborns. Despite decades of research, the molecular mechanism underlying its pathogenesis remains unclear. Many studies have shown that preterm birth is associated with health risks across the later life course. The “fetal origins” hypothesis postulates that adverse intrauterine exposures are associated with later disease susceptibility. Our recent studies have focused on the placental epigenome at term. We extended these studies to genome-wide placental DNA methylation across a wide range of gestational ages. We applied methylation dependent immunoprecipitation/DNA sequencing (MeDIP-seq) to 9 placentas with gestational age from 25 weeks to term to identify differentially methylated regions (DMRs).

**Results:**

Enrichment analysis revealed 427 DMRs with nominally significant differences in methylation between preterm and term placentas (*p* < 0.01) and 21 statistically significant DMRs after multiple comparison correction (FDR *p* < 0.05), of which 62% were hypo-methylated in preterm placentas vs term placentas. The majority of DMRs were in distal intergenic regions and introns. Significantly enriched pathways identified by Ingenuity Pathway Analysis (IPA) included Citrulline-Nitric Oxide Cycle and Fcy Receptor Mediated Phagocytosis in macrophages. The DMR gene set overlapped placental gene expression data, genes and pathways associated evolutionarily with preterm birth.

**Conclusion:**

These studies form the basis for future studies on the epigenetics of preterm birth, “fetal programming” and the impact of environment exposures on this important clinical challenge.

**Electronic supplementary material:**

The online version of this article (10.1186/s12881-019-0835-6) contains supplementary material, which is available to authorized users.

## Background

Despite decades of research, the underlying cause of preterm birth remains enigmatic. It is a leading cause of newborn morbidity, hospitalization, and developmental delays [1]. In addition, preterm birth is associated with health risks across the later life course of the newborn, including cardiovascular disease, metabolic syndromes, psychiatric conditions, obesity and cognitive disabilities [1, 2]. The “fetal origins” or Developmental Origins and Health and Disease (DOHaD) hypothesis, developed from a series of epidemiologic observations, demonstrated that measures of birth size were associated with long-term chronic disease risk [3]. Numerous investigations have shown that antenatal maternal environmental factors, including diet, xenobiotic exposure, stress, and lifestyle factors can alter fetal growth and result in permanent biological and physiologic changes of the offspring [3]. Environmental factors like race, diet, smoking, socioeconomic status may also increase the risk of spontaneous preterm birth [1, 4, 5] and are associated with epigenetic alterations [6].

DNA methylation is the most well studied epigenetic mechanism of gene regulation, often associated with transcriptional silencing of downstream gene(s). The presence of the methyl group(s) alone is not sufficient for transcriptional silencing, but instead alters recruitment of component proteins related to gene repression and results in a silenced chromatin conformation. DNA methylation is an essential epigenetic mechanism in fetal development [7].

The placenta facilitates the exchange of gas, nutrients, and waste between the mother and the fetus, and modulates effects on the fetus from the mother’s immune system, thus playing an essential role in fetal growth and development. It is also essential in understanding the long-term effects of in-utero development on post-natal disease. The placenta undergoes many changes throughout gestation and the mechanisms behind these changes need to be better understood. In an attempt to do so, several studies have examined genome wide expression differences in placentas at different time points during gestation, comparing first, second and third trimester placental methylation [8, 9]. Changes in expression with increasing gestational age were found in common between the studies. Others are attempting to better understand placental development and fetal programming through the study of epigenetic factors, including DNA methylation of placental tissue and umbilical cord blood. Studies of umbilical cord blood from preterm and term pregnancies have releaved differences in methylation associated with gestational age [10, 11]. Novakovic et al. have studied genome scale placental promoter methylation from the three trimesters of pregnancy, revealing a progressive increase in methylation from first to third trimester. They also identified increased inter-individual variability in third trimester samples [12]. Other studies have alsofound varied methylation differences associated with gestational age comparing placentas in the third trimester, as well as a global increase in methylation with gestational age (28–40 weeks) [13–15]. In addition, the placenta has the highest overall variability in DNA methylation when compared to other tissues [16]. These studies all support the emerging paradigm that the placenta is an active mediator of fetal well-being and neurodevelopmental outcome and can serve as a blueprint for intrauterine life [17]. This is an exploratory study seeking to investigate genome wide placental DNA methylation across a wide range of preterm gestational ages and compared it to that of placenta from term deliveries. In order to generate genome-wide information, we employed immunoprecipitation of methylated DNA followed by whole-genome sequencing, so called MeDIP-seq [18]. We hypothesize that using this approach, we would be able to identify potential regions of interest and pathways involved in and influenced by changes in placental methylation associated with preterm birth and gestational age. Our objectives were to demonstrate the feasibility of this approach and to generate placental methylation data that would be useful to our own studies and to those of others.

## Results

### Placental sample and patient characteristics

Placental samples of villous parenchyma were taken from four quadrants between the chorionic and basal plate. Table 1 shows summary clinical characteristics of the cohort of placental samples and the associated patients. Placental samples were obtained from six preterm pregnancies (gestational age 25–34 weeks) and three term pregnancies (37–41 weeks). The average birth weights of the fetuses were 1541 g vs 3033 g and the average gestational ages were 30 weeks vs 39 weeks, respectively. We also recorded maternal pregnancy factors including BMI, but the variance was large and thus the means were not significantly different between the two groups. All fetuses had birth weights that were appropriate for gestational age. Among placentas from the preterm pregnancies, two of the mothers were diagnosed with some degree of hypertension. There was no history of drug use. One mother, who delivered preterm, admitted to smoking during pregnancy. Detailed clinical data for each sample can be found in Additional file 1.

**Table 1:** Summary of Clinical characteristics of sampled patients

Clinical Data	Cases (*n* = 6)	Controls (*n* = 3)	*P*-value
Gestational Age (avg weeks)	30	39	0.001
Maternal BMI (avg kg)	29.5	40.7	0.32
Birth Weight (avg g)	1541	3033	0.001
Drug Use (number of samples)	0	0	–
Smoking (number of samples)	1	0	–
Preeclampsia (number of samples)	2	0	–
Male Sex of Infant (number of samples)	3	2	–
Race/Ethnicity (number of samples)	White (5) Other (1)	White (2) Hispanic (1)	–

### Differentially methylated regions (DMR) associated with preterm birth

**Fig. 1:**
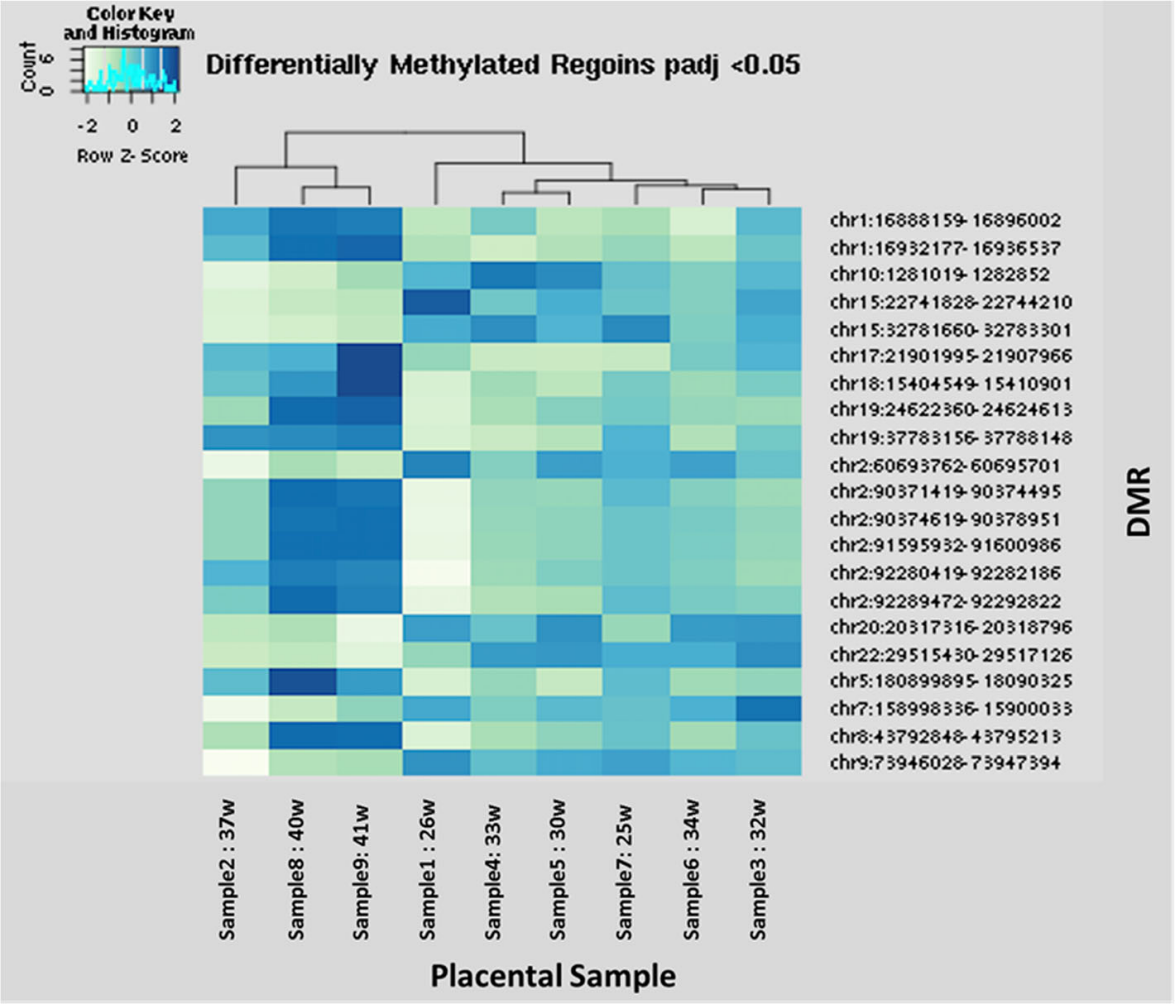
Peak Intensity heat map: A heat map of the read counts of the 21 DMRs for each of the 9 samples. Samples 2, 8 and 9 are the term placentae, and the remaining is preterm. Unsupervised clustering was used to order the columns. Darker blue squares represent more reads/higher methylation whereas lighter green squares represent less reads/lower methylation

We used the bioinformatics tools DiffBind and DESeq2 to test for association with preterm birth using methylation peak counts as the outcome and PTB status as the independent variable. The raw zipped fastq files and the peak count matrix have all be uploaded to GEO and can be found with the following accession number: *GSE120458 (**https://www.ncbi.nlm.nih.gov/geo/query/acc.cgi?acc=GSE120458**)*. We found 427 peaks with nominally significant differences in methylation between cases and controls, (*p* < 0.01) [see Additional file 2]. Following FDR correction, there were 21 DMRs that associate with PTB using a filter for low mean counts to maximize the number of FDR significant peaks at an adjusted *p* < 0.05. These 21 significant DMRs and their annotations are shown in Table 2. The peak heights (read counts) of the 21 DMRs associated with PTB are also visualized in a heat map in which unsupervised clustering was used to group the patients (columns) (Fig. 1). The three term patients (Samples 2, 8 and 9) cluster together and are distinct from the 6 preterm samples. Among the 21 DMRs associated with PTB, 62% were hypo-methylated in preterm placentas compared to term placentas. Similar percentages were found for the uncorrected significant DMRs. We next used the R Bioconductor package CHipSeeker [19] to annotate the DMRs associated with PTB with their nearest gene. The 427 regions are associated to 342 unique genes. The highest percentage of DMRs map to distal *intergenic* regions (57.38%) followed by *introns*, other than the first intron and promoter regions. A larger percentage of DMRs were located in proximal promoter regions (< 1 kb upstream) compared to more distal regions (> 2-3 kb followed by 1-2 kb upstream) (see Additional file 3).

**Table 2:** Annotated DMR’s Associated with Preterm Birth

DMR Location	DMR Width	baseMean	log2 FoldChange	padj	Annotation	Nearest Gene
chr10:1281019–1,282,852	1833	614.6687	−1.39475	0.001099	Intron	ADARB2
chr22:29515430–29,517,126	1696	2125.015	−0.7487	0.001099	Intron	KREMEN1
chr2:60693762–60,695,701	1939	293.8868	−0.96653	0.003059	Intron	BCL11A
chr1:16888159–16,896,002	7843	1691.305	0.664717	0.018052	3′ UTR	MIR3675
chr15:22741828–22,744,210	2382	1025.047	−1.02204	0.018052	Exon	GOLGA6L1
chr15:32781660–32,783,301	1641	391.2485	−0.56857	0.018052	Distal Intergenic	GOLGA8O
chr17:21901995–21,907,966	5971	1435.135	1.262002	0.018052	Promoter (<=1 kb)	FLJ36000
chr19:24622360–24,624,613	2253	338.0393	1.234145	0.018052	Distal Intergenic	HAVCR1P1
chr19:37783156–37,788,148	4992	786.5645	1.768082	0.018052	Distal Intergenic	HKR1
chr2:92280419–92,282,186	1767	1109.498	1.027544	0.018052	Distal Intergenic	ACTR3BP2
chr20:20317316–20,318,796	1480	713.2818	−0.66627	0.018052	Intron	INSM1
chr9:73946028–73,947,394	1366	290.2041	−0.64868	0.024388	Intron	TRPM3
chr18:15404549–15,410,901	6352	972.9929	1.687868	0.02956	Distal Intergenic	LOC644669
chr2:92289472–92,292,822	3350	4738.836	1.060257	0.032703	Distal Intergenic	ACTR3BP2
chr1:16932177–16,936,537	4360	710.057	0.489202	0.048809	5′ UTR	NBPF1
chr2:90371419–90,374,495	3076	1492.051	1.021356	0.048809	Intron	MIR4436A
chr2:90374619–90,378,951	4332	2470.989	1.010006	0.048809	Intron	MIR4436A
chr2:91595932–91,600,986	5054	2660.109	0.993924	0.048809	Distal Intergenic	LOC654342
chr5:180899895–180,903,257	3362	290.8312	0.778464	0.048809	Distal Intergenic	OR4F16
chr7:158998336–159,000,338	2002	314.5755	−0.72705	0.048809	Distal Intergenic	VIPR2
chr8:43792848–43,795,213	2365	1959.954	1.112517	0.048809	Distal Intergenic	POTEA

**Fig. 2:**
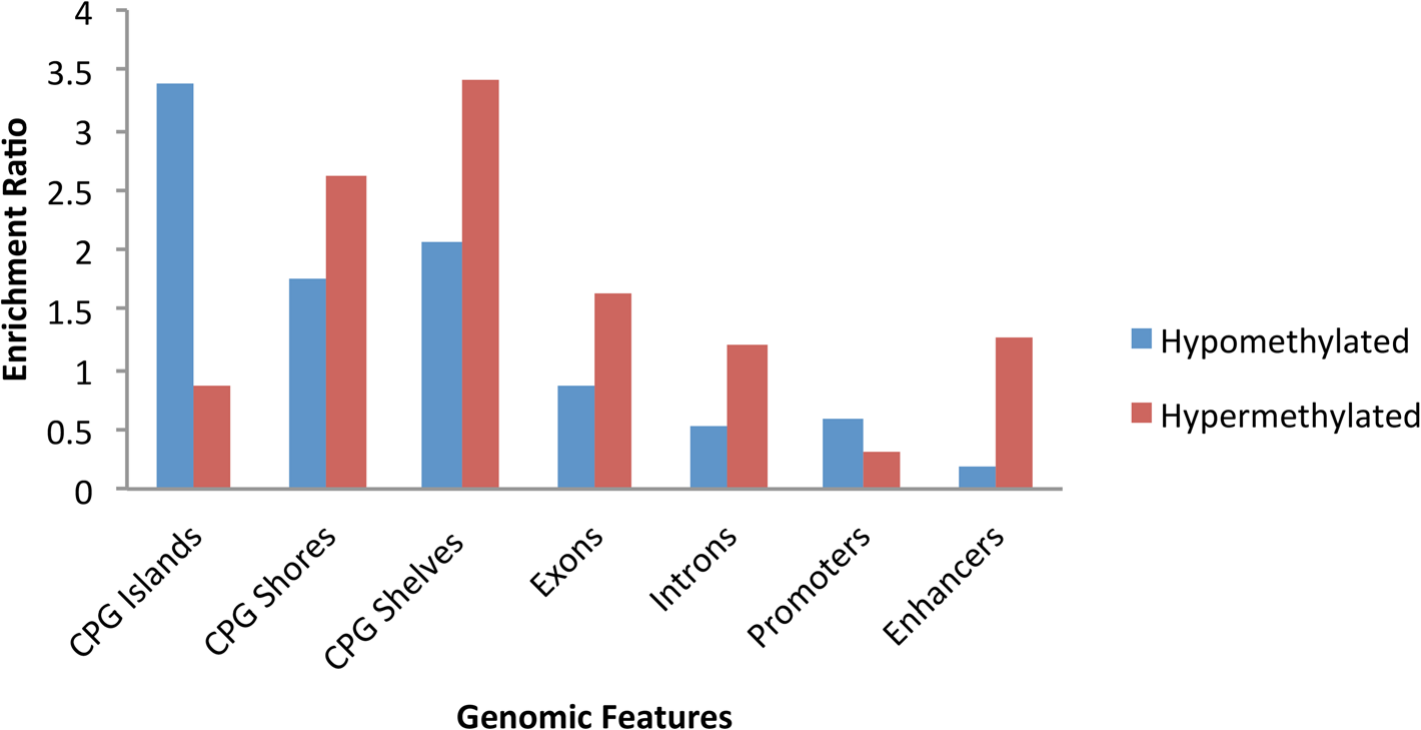
Enrichment of genomic features amongst differentially methylated regions: Genomic feature enrichment for hyper-methylated (left) and hypo-methylated (right) DMRs. Introns, Exons and CpG islands were obtained from UCSC Genome Browser and shores and shelves are defined as 2 kb and 4 kb up and down stream of the islands. Promoters and Enhancers were obtained from Roadmap Epigenome Placental cell line

Enrichment scores for a variety of genomic features for the hyper-methylated and the hypo-methylated DMRs independently are shown in Fig. 2. The hypo-methylated DMRs were enriched for CpG Islands and the hyper-methylated regions were enriched for CpG shores and shelves.

### Differentially methylated regions (DMR) are associated with gestational age

We used the same pipeline to test for association of DNA methylation with preterm birth using methylation peak counts as the outcome and gestational age in weeks as a continuous, independent variable. We found 667 peaks with nominally significant differences in methylation between cases and controls, (*p* < 0.01) [see Additional file 4]. Following FDR correction, we found 67 significant DMRs that associate with gestational age, using a filter for low mean counts to maximize the number of FDR significant peaks at an adjusted *p* < 0.05. Table 3 contains these 67 DMR and their annotations. The percentages of the DMRs that map to the genomic annotation categories are almost identical to the comparison between preterm birth and term.

**Table 3:** Annotated DMR’s Associated with Gestational Age

DMR Location	DMR Width	baseMean	log2FoldChange	padj	Annotation	Nearest Gene
chr11:1069274–1,070,940	1666	275.32	−0.077895692	0.000669	Distal Intergenic	MUC2
chr7:158509261–158,511,170	1909	785.4202	−0.055129353	0.001379	Distal Intergenic	NCAPG2
chr8:143302532–143,305,491	2959	708.6988	−0.057666861	0.001379	Intron	LINC00051
chr11:51578780–51,581,392	2612	5862.829	0.12089555	0.007632	Distal Intergenic	OR4C46
chr15:22741828–22,744,210	2382	1025.047	−0.091557436	0.007632	Exon	GOLGA6L1
chr17:21901995–21,907,966	5971	1435.135	0.114589697	0.008451	Promoter (<=1 kb)	FLJ36000
chr11:51587551–51,593,541	5990	6613.759	0.114797282	0.012877	Distal Intergenic	OR4C46
chr10:115540476–115,542,108	1632	369.5293	−0.058238042	0.015459	Promoter (2-3 kb)	MIR4483
chr4:9875–10,674	799	300.733	−0.066430834	0.020835	Distal Intergenic	ZNF595
chr5:49415136–49,417,649	2513	629.6273	0.098693759	0.020835	Distal Intergenic	EMB
chr15:56071747–56,073,363	1616	280.2815	−0.044766281	0.028409	Distal Intergenic	PRTG
chrY:59027085–59,033,404	6319	757.9891	0.085296918	0.028937	Distal Intergenic	SPRY3
chr10:127579482–127,584,720	5238	732.2817	0.072848407	0.032657	Promoter (<=1 kb)	DHX32
chr10:134878560–134,880,844	2284	289.1369	−0.080610086	0.032657	Distal Intergenic	ADGRA1
chr11:1795935–1,798,177	2242	452.7534	−0.059025405	0.032657	Distal Intergenic	MOB2
chr11:51570928–51,573,041	2113	623.7097	0.10472301	0.032657	Distal Intergenic	OR4C46
chr11:51581774–51,585,209	3435	3634.642	0.099090126	0.032657	Distal Intergenic	OR4C46
chr19:24622360–24,624,613	2253	338.0393	0.100239042	0.032657	Distal Intergenic	HAVCR1P1
chr2:60693762–60,695,701	1939	293.8868	−0.074300347	0.032657	Intron	BCL11A
chr2:90374619–90,378,951	4332	2470.989	0.08904097	0.032657	Intron	MIR4436A
chr2:92280419–92,282,186	1767	1109.498	0.085845619	0.032657	Distal Intergenic	ACTR3BP2
chr21:11121793–11,128,301	6508	1510.744	0.043743747	0.032657	Distal Intergenic	BAGE
chr5:49413369–49,415,026	1657	555.3467	0.102896519	0.032657	Distal Intergenic	EMB
chr6:58775746–58,780,286	4540	59,219.18	0.088992793	0.032657	Distal Intergenic	GUSBP4
chr6:132921367–132,922,856	1489	702.8588	−0.077101273	0.032657	Distal Intergenic	TAAR3
chr7:155125413–155,128,850	3437	1162.764	−0.038748136	0.032657	Distal Intergenic	INSIG1
chr7:155199140–155,201,882	2742	355.5753	−0.077494132	0.032657	Distal Intergenic	EN2
chr2:91603906–91,606,341	2435	1002.445	0.094259612	0.033077	Distal Intergenic	LOC654342
chr1:16888159–16,896,002	7843	1691.305	0.055571393	0.035079	3′ UTR	MIR3675
chr2:91595932–91,600,986	5054	2660.109	0.085847679	0.035079	Distal Intergenic	LOC654342
chr5:49428377–49,432,607	4230	1837.6	0.094529868	0.035079	Distal Intergenic	EMB
chr5:49434812–49,441,568	6756	3582.595	0.092033374	0.035079	Distal Intergenic	EMB
chr8:143093456–143,095,020	1564	434.2594	−0.048625159	0.037056	Distal Intergenic	MIR4472–1
chr9:43157894–43,160,792	2898	440.6311	−0.051599615	0.038038	Distal Intergenic	LOC642929
chr3:196625149–196,626,329	1180	5459.852	0.091127805	0.03925	Intron	SENP5
chr1:2775172–2,776,643	1471	300.3145	−0.049869539	0.043507	Distal Intergenic	TTC34
chr1:161411315–161,417,356	6041	980.0181	0.059054185	0.043507	Exon	FCGR2A
chr1:227165108–227,167,121	2013	321.0379	−0.05472419	0.043507	Promoter (<=1 kb)	ADCK3
chr10:42639382–42,642,799	3417	471.9378	0.053591881	0.043507	Distal Intergenic	LOC441666
chr12:117759233–117,761,187	1954	733.3737	−0.051097394	0.043507	Intron	NOS1
chr12:131743021–131,745,096	2075	402.1728	−0.04241032	0.043507	Distal Intergenic	LINC01257
chr14:104680716–104,682,479	1763	393.9317	−0.039036454	0.043507	Distal Intergenic	KIF26A
chr14:106130890–106,133,431	2541	327.5869	−0.050328469	0.043507	Intron	ELK2AP
chr18:9876–11,028	1152	869.3626	−0.058790034	0.043507	Distal Intergenic	ROCK1P1
chr2:90380982–90,382,232	1250	703.058	0.087608099	0.043507	Intron	MIR4436A
chr2:90390888–90,393,740	2852	1224.857	0.082639282	0.043507	Intron	MIR4436A
chr2:232245135–232,247,014	1879	684.5341	−0.049273959	0.043507	Distal Intergenic	B3GNT7
chr2:233878888–233,880,783	1895	458.8731	−0.042533652	0.043507	Promoter (<=1 kb)	NGEF
chr21:47233703–47,236,436	2733	660.0114	−0.045879449	0.043507	Intron	LOC100129027
chr22:28043663–28,045,838	2175	319.4394	−0.045382128	0.043507	Distal Intergenic	MN1
chr3:185842547–185,844,972	2425	338.0063	−0.040820001	0.043507	Distal Intergenic	ETV5
chr4:3679282–3,681,125	1843	632.2126	−0.039394589	0.043507	Promoter (<=1 kb)	LOC100133461
chr5:171997237–171,998,674	1437	574.0105	−0.040752286	0.043507	Distal Intergenic	NEURL1B
chr5:172145042–172,146,642	1600	283.15	−0.039405643	0.043507	Distal Intergenic	DUSP1
chr7:35083300–35,086,409	3109	667.0347	−0.063938851	0.043507	Exon	DPY19L1
chr8:27426562–27,428,394	1832	618.5586	−0.046453345	0.043507	Distal Intergenic	CLU
chrX:148615982–148,617,887	1905	496.8346	−0.071187463	0.043507	Promoter (<=1 kb)	IDS
chr14:77322208–77,324,017	1809	358.4726	−0.036853715	0.043857	Exon	LRRC74A
chr14:94213175–94,214,970	1795	274.9046	−0.044559973	0.043963	Intron	PRIMA1
chr8:143824284–143,827,190	2906	872.6112	−0.056328342	0.043963	Promoter (<=1 kb)	SLURP1
chr2:92289472–92,292,822	3350	4738.836	0.085456146	0.04533	Distal Intergenic	ACTR3BP2
chr1:15170988–15,172,589	1601	341.8562	−0.043070132	0.045985	Intron	KAZN
chr1:22873178–22,875,162	1984	628.9255	−0.048195935	0.045985	Distal Intergenic	EPHA8
chr15:32781660–32,783,301	1641	391.2485	−0.044570446	0.045985	Distal Intergenic	GOLGA8O
chr2:92294963–92,300,499	5536	4633.964	0.085605878	0.048908	Distal Intergenic	ACTR3BP2
chr4:5852906–5,854,271	1365	315.8726	−0.042689188	0.048908	Exon	CRMP1
chr7:15223273–15,225,157	1884	555.7869	−0.050035386	0.048908	Distal Intergenic	DGKB

In an attempt to distinguish DMRs that are solely a result of gestational timing from those which could be explained by experience dependent alterations, we looked for overlap and differences between the continuous analysis on gestation age and the categorical analysis on PTB status. Ten out of the 21 DMRs show methylation changes that are associated to both PTB and gestational age. The remaining 11 DMRs may reflect changes due to experience dependent alterations. Additionally, 215 DMRs were found significant in both the dichotomous and continuous models (*p*-value <.01), mapping to 177 unique genes.

### Comparative gene set analysis

To enhance discovery and interpretation of these findings, we compared our DMRs and their nearest annotated genes with previously established gene sets that have been shown to be associated with preterm birth and pregnancy. We compared the genes nearest to the DMRs associated with PTB and gestational age to transcription profiles from preterm and term placenta samples [20]. The results, shown in Tables 4 and 5, are for genes that were upregulated and downregulated, respectively. This table also shows the genes nearest DMRs that are contained within a set of genes that are in networks and pathways related to preterm birth, outlined in the Database for Preterm Birth (dbPTB) [21]. Lastly, we compared the genes nearest our DMRs to a set of genes that have been previously found by Lynch et al. to be uniquely expressed in the endometrium of placental mammals and shown to be important in the evolution of pregnancy [22]. While the number of DMR associated genes overlapping each of these preterm birth gene sets is greater than the number expected by chance, this comparison was not statistically significant.

**Table 4:** Comparative Analysis of Nearest Genes to DMRs associated with PTB

Overexpressed in PTB Placenta [20]	Under expressed in PTB Placenta [20]	Mammalian Gain of Function [22]	Mammalian Loss of Function [22]	dbPTB Curated Gene Set [21]
**TFRC** NBPF10 JAM3 ARHGEF7 DAPK1	GUSBP1 MFSD2A PSPC1	KREMEN1 **TFRC** DPY19L1 C1orf159 K1F14 NEDD4L C1QTNF3 EDIL3 PSD3 ARPC5L	C15orf27 PRSS33 CAPN13 NPBWR2 MYOM2	ATRNL1 NOS1

**Table 5:** Comparative Analysis of Nearest Genes to DMRs associated with Gestational Age

Overexpressed in PTB Placenta [20]	Under expressed in PTB Placenta [20]	Mammalian Gain of Function [22]	Mammalian Loss of Function [22]	dbPTB Curated Gene Set [21]
JAM3	PRKAG2 MFSD2A PSPC1 BCL2	DPY19L1 TMEM132C C1orf159 K1F14 AKAP6 ZNF532 CMPK2 SLC8A1 ADCY2 C1QTNF3 KHDRBS3	FERMT1 L1F1A CAMK2A KCNQ2 RD3 **MBL2** PRSS33 GSG1L EPHB1 PDE6B CLVS2 MYOM2	CDC25A COL1A2 COL5A1 ETV5 F13A1 GC HS3ST3A1 IL1R2 KATNAL2 LOXHD1 **MBL2** MYH9 NOS1 SMAD6 SOX17 BEAN1 KCNN3

Nonetheless, it is of interest that a hyper-methylated DMR associated with PTB is in nearest proximity to the *TFRC* gene *(Transferrin Receptor 1),* which is associated with prematurity in placental transcription profiles and evolutionarily conserved endometrial genes. TFRC is an essential protein for iron transfer across the placenta and changes in its expression have been associated with IUGR and preeclampsia. In addition, a hypo-methylated DMR is associated with gestation age in the MLB2 (mannose binding lectin) gene, which overlaps the latter two comparative gene sets. MLB2 codes for a protein which plays a role in fetal inflammatory response to infection and injury.

### Pathway analysis

Given that PTB is not a monogenic disorder, we were interested in the pathways associated with the genes neared to the above DMRs. We reasoned this might provide insight into the functional context of the PTB associated DMRs. We found 9 canonical pathways significantly enriched in the PTB associated DMRs (−log *p*-value > 1.3) The most significant pathways included Superpathway of Citrulline Metabolism, Citrulline-Nitric Oxide Cycle, Fc-gamma Receptor Mediated Phagocytosis in Macrophages and the Urea Cycle. The Citrulline Metabolism pathway and the Citrulline Nitric Oxide Cycle pathway contain NOS1 (nitric oxide synthase 1) which has a proximal hyper-methylated DMR associated with both PTB and gestational age and also in the dbPTB gene set of networks and pathways related to PTB [21]. IPA also returned the top 25 gene interaction networks. The top scoring networks contained 25 genes from our DMR gene set and were related to cell death, organismal survival and gene expression.

We did additional pathway analysis on the set 177 unique genes nearest the 215 DMRs which were significant in both models and on the set of genes nearest the DMRs only significant in the PTB model. The aforementioned pathways remain the most significant ones. For the DMRs which were significant for both models, Superpathway of Citrulline Metabolism, Citrulline-Nitric Oxide Cycle, and the Urea Cycle were the top canonical pathways. For the DMRs only significant in the PTB model, Fc-gamma Receptor Mediated Phagocytosis in Macrophages remained in the top list of pathways, in addition to some new pathways: Integrin signaling, DNA damage signaling and FAK signaling.

## Discussion

We used methylation-dependent immunoprecipitation followed by high throughput sequencing to generate non-biased, genome-wide map of DNA methylation in placenta from a wide range of gestational ages. We investigated regions for which there was differential methylation between preterm (< 34 weeks) and term placentas (> 37 weeks), as well as regions for which the differences in methylation were associated with the continuous variable gestational age. Our results demonstrate significant differences in DNA methylation in preterm versus term placenta. Approximately half of the DMRs associated with preterm birth were not significantly associated with changes in gestational age. There were more hypo-methylated regions in preterm patients compared to term patients. The highest percentages of differentially methylated regions mapped to distal intergenic regions followed by introns, exons and then promoter regions. Mapping of these significant DMRs to the nearest genes demonstrated some overlap with patterns of differential gene expression in placentas from preterm and term patients [20]. There was also overlap with genes shown to be evolutionarily linked to preterm birth and to networks and pathways associated with preterm birth [21, 22].

Both candidate gene studies and genome-wide studies of DNA methylation in the placenta have been performed to investigate the mechanism(s) of preterm birth. One study found a positive association between global methylation and gestational age but others found little variation amongst the partially methylated domains across all three trimesters [11, 14]. Another study of promoter region methylation found overall differences in methylation between second and third trimester placentas, but not between first and second trimester [12]. Several studies examining gestational age and DNA methylation used umbilical cord blood to gain understanding into fetal programming and methylation state at birth. In one study, among the 39 genes showing differential methylation, 29 showed a decrease in methylation with increase in gestational age while the remainder showed an increase and no relationship to type of delivery [11]. Parets et al. studied methylation of cord blood leukocytes from 24 weeks to 41 weeks [10]. Most sites showed lower degrees of methylation with shorter gestational age, suggesting that one mechanism regulating the extent of methylation is gestational timing. We and others have also found associations with the preterm birth process itself. The Norwegian Mother and Child Cohort Study (MoBa) compared cord blood methylation with birthweight and found both increased and decreased patterns of methylation associated with specific genes [23]. Another study using the Illumina 450 k array found 1400 variably-methylated regions which correlated with significant variables in the intrauterine environment including maternal smoking, maternal depression, maternal BMI, infant birthweight and gestational age [24]. Thus, while no unifying picture of the association between gestational age and DNA methylation has been demonstrated, we believe the mechanisms regulating the extent and pattern of placental DNA methylation include programmed changes linked to gestational timing as well as experiential changes. Our study, with a wide range of gestation ages, using a non-biased, genome-wide approach, shows a significant effect of both gestational age as a continuous predictor and PTB status as a categorical predictor of placental DNA methylation.

The site of methylation may be crucial to the effect on gene expression or a reflection of the impact of environment on gene expression. Clusters of CpG’s also known as CpG islands (CGI) are present in 5′ promoter regions of many genes. Methylation can also take place in shores and shelves, which are more distant to the promoter. Some studies have shown that tissue- and cancer-specific DMRs occur more frequently within CpG shores than CGIs themselves [25]. The functional implications of alterations in methylation are context-specific. Methylation in the immediate vicinity of the transcription start site is believed to block initiation, whereas methylation in the gene body may stimulate transcription elongation and/or have an impact on splicing [25]. We saw the greatest degree of differential methylation (almost 60%) in distal intergenic regions. Second greatest differential methylation was seen in introns other than the first intron. In addition, enrichment analysis showed that hypo-methylated DMRs were enriched for CpG Islands, while hyper-methylated DMR were enriched for CpG shores and shelves (Fig. 2). The annotation results, along with the later enrichment results, are consistent with the results from previous studies suggesting methylation is more dynamic outside of CpG islands in promoter regions. The enrichment of CpG islands amongst the hypo-methylated DMRs could be linked to chromosomal instability and imprinting [26]. The implications of the intergenic and intragenic methylation, as well as in shores and shelves on preterm birth are significant, yet mechanistically still unclear.

The most significant pathway associated with the genes nearest to the 427 DMRs we observed was Citrulline-Nitric Oxide Cycle, which contains the NOS1 gene. Our results found a hypermethylated DMR associated with both PTB and gestational age proximal to NOS1. NO is secreted by placenta [27] and known to modulate both fetal and utero placental blood flow [28]. Bielecki et al. found a lower concentration of NO in a group of women with premature contractile activity in comparison with gestational age-matched healthy pregnant women [29]. In another study the amniotic fluid concentration of NO was significantly higher in patients with intra-amniotic infection compared to those without intra-amniotic infection [30]. A decrease in NO production may contribute to the initiation of labor and cervical ripening [31]. A study suggests that NO produced by the placenta could play role in maintaining uterine quiescence by paracrine effect [32]. These results suggest that increased methylation of NOS1 may play an important role in the production of NO and subsequently preterm birth.

Another significant pathway was Fc-gamma Receptor Mediated Phagocytosis in Macrophages. There is abundant evidence for Fc gamma R mediated transcytosis of IgG in the placenta. The transfer of IgG from mother to fetus begins around 13 weeks of gestation and the total IgG concentrations in newborns is directly related to length of gestation. Infants born preterm have substantially lower IgG levels than full-term babies [33]. We also identified a DMR whose nearest gene is mannose binding lectin (*MBL2),* which has previously been identified by pathway and network analysis to be related to preterm birth and evolutionarily associated as well [21, 22]. *MBL2,* found in amniotic fluid, is a serum protein involved in the activation of the complement system of the innate immune system and plays a role in fetal inflammatory response to infection and injury [34, 35]. It activates complement system by binding to carbohydrates, present on a wide range of proteins [36]. Moreover, fetal *MBL2* haplotypes and in utero exposure to viral infection increases the risk of preterm birth [37].

When we compared our DMR results to data sets important in preterm birth, we identified a hyper-methylated peak whose nearest gene is transferrin receptor 1, *TFRC*. *TFRC* is expressed in the placenta and mediates cellular iron uptake. Iron deficiency during pregnancy increases the risk of preterm birth [38]. While *TFRC* was upregulated spontaneous preterm birth in the Chim et al. placental expression study, it was also upregulated in the Lynch evolution of mammalian pregnancy and found to be reduced placentas with intrauterine growth restriction and preeclampsia [39]. Because prematurity, IUGR and preeclampsia have different pathogenic etiologies, the results suggest the importance of further investigation of the epigenetic regulation of TFRC with respect to pregnancy related disorders.

The current study demonstrates the feasibility of sample collection, technical analysis and data processing. Potential limitations of the study are the relatively small sample size and the diversity of patients. Nonetheless, in order to clearly define an effect of prematurity, we purposefully collected placental samples from a wide range of gestational ages. There was some variation in the mothers’ clinical features beyond prematurity that may have impacted DNA methylation. Nonetheless, these unbiased data provide a useful reference for future studies by us and others. In addition, we chose to study genome-wide methylation using MeDIP-Seq due to its feasibility and moderate expense as compared to other techniques such as Whole Genome Bisulfite Sequencing. The affinity-based approach coupled with deep sequencing has a resolution of 100-300 bp and is cost effective when single-base resolution is not necessary [40, 41]. Previous research suggested that at 1x coverage, a majority of the methylated CpG can be studied [40]. It is important to note that MeDIP-seq, similar to restriction enzyme digestion approaches, can only measure relative enrichment of methylated DNA rather than absolute methylation levels. Lastly, another advantage of MeDIP-seq over WGBS is its ability to detect both 5-Methylcytosine (5mC) and 5-hydroxymethylctyosine (5hmC) independently [40, 41].

## Conclusions

We identified associations between DNA methylation and preterm birth, building on recent findings that prenatal environmental exposures mediate developmental programming effects through epigenetic changes [3, 42]. Our data demonstrate that in future studies it will be important to include gestational age matched samples with prenatal conditions like intrauterine growth restriction and environmental exposures such as drug use, environmental toxins and intrauterine infection. This will allow us to predict which local differences in methylation segregate with which combinations of phenotype. In addition, future studies should compare gestational age matched placentas from births due elective cesarean (before the onset of labor). These studies form the basis for future studies on the epigenetics of preterm birth, “fetal programming” and the impact of environment exposures on this important clinical challenge.

## Methods

### Placental samples

Placenta samples were collected by our research staff at Women & Infants Hospital of Rhode Island. They obtained shortly after delivery from births ranging from 25 weeks to 41 weeks of gestational age. Samples of villous parenchyma were taken from four quadrants between the chorionic and basal plate. Care was taken to avoid maternal decidua and areas of hemorrhage or calcification. Samples were placed immediately into RNAlater^*Tm*^ (Ambion, Inc., #AM7021) and stored at − 80 °C until DNA extraction. Preliminary studies have shown that macromolecules like RNA levels were similar from each sample site and that this approach was equal to or superior to immediate immersion in liquid nitrogen for prevention of RNA degradation [43, 44].

### DNA extraction

Genomic DNA was extracted using the Qiagen DNeasy Blood and Tissue kit (Qiagen, # 69506) and quantified on a NanoDrop 1000. 5μg of DNA was digested to fragment size 200–300 base pairs using dsDNA Fragmentase enzyme at 37 °C for 30 min (New England Biolabs, #MO348L). Fragments were end-repaired, 3′-ends were adenylated, and appropriate adapter indexes were ligated using the Truseq protocol (Illumina). Between each reaction, fragments were cleaned using Agencourt AmPure magnetic beads (Beckman Coulter, # A63881). Fragments were then amplified by PCR at 98 °C/30 s; 10 cycles of 98 °C/10s, 60 °C/30s, 72 °C/30s; and 72 °C 5 min with a hold at 10 °C. Enriched fragments were then cleaned using Agencourt AmPure magnetic beads and quantified before methylation-dependent immunoprecipitation.

### MeDIP-seq

Methylated-DNA immunoprecipitation was performed using the Methylated-DNA IP kit (Zymo Research, # D5101). 320 ng of each sample was mixed with denaturation buffer and heated to 98 °C for 5 min. DNA is then mixed with MIB buffer, ZymoMag Protein A beads, and Mouse Anti-5-Methylcytosine from and incubated at 37 °C for one hour, with mixing every 15 min. The tubes were rocked, allowed to cluster, washed with reagent buffer and then eluted at 75 °C for 5 min. This was followed by a 2-min spin in a mini centrifuge at 18,000 g. The recovered DNA underwent 100 bp paired-end sequencing in the Brown University Genomics Core in triplicate on an Illumina HiSeq 2500.

Raw sequence reads were separated according to sample-specific barcodes and mapped to the NCBI Build UCSC Hg19 human genome using the Burrows-Wheeler Aligner (BWA v0.6.2) [45]. The SAM files were converted to BAM files with SamTools (v0.1.18) [46] and duplicate reads (reads with the same start location) were removed using Picard Tools (v1.77) (https://github.com/broadinstitute/picard). We used Model-based Analysis for ChIP-Seq (MACS v1.4) [47] to identify significantly enriched regions (peaks) using *p* < 1 × 10^− 5^ as the significance threshold for each individual and technical replicate independently.

### Identification of differentially methylated regions

We used the R Bioconductor packages DiffBind (http://bioconductor.org/packages/DiffBind/) and DESeq2 [48] to identify Differentially Methylated Regions (DMRs). We used DiffBind to identify a peak set for the study cohort, requiring that each individual’s consensus peak set contain only peaks which were present in all three technical replicates. For each individual, the read count for each peak in the consensus peak set was merged by taking the sum over all three technical replicates. DMRs were identified using DESeq2. *P*-values were corrected using FDR with independent filtering of overall low mean counts.

### Genomic annotation and enrichment

DMRs with a *p*-value < 0.01 were annotated using R Bioconductor package ChIPseeker [19] to retrieve the nearest gene to the peaks of interest and annotate the genomic region of the peak. CpG islands and Refseq gene exons and introns were downloaded from the UCSC Genome Browser [49]. CpG shores and shelves were defined 2 kb and 4 kb up and downstream from the CpG islands, respectively. The Hg19 reference genome was spilt into 500 bp windows and each window was annotated with the above genomic features if any overlap existed. The ChromHMM annotation of the Placenta Cell Line from the Roadmap Epigenome Project, obtained from the UCSC Genome Browser, was used to align the 500 bp windows with “promoter” and “enhancer” state annotation [50]. The enrichment score for each genomic feature (CpG islands, shores, shelves, exons, introns, promoters, and enhancers) with respect to the DMRs was calculated via the method in Zhang et al. as the ratio between the fraction of DMRs overlapping widows with genomic feature and the fraction of total windows with the genomic feature [51].

### Comparative gene set analysis

In order to examine the potential role of DNA methylation in the regulation of preterm birth we compared our DMRs with previously published gene sets associated with preterm birth and pregnancy.

Chim et al. used an array based approach to study differential placental gene expression between spontaneous preterm birth and spontaneous term birth. “They reported 240 significantly upregulated and 186 significantly downregulated genes in the placenta associated with spontaneous preterm birth.” [20]. We also compared the significant DMRs with a gene set identified in curated articles, networks and pathways important in the risk of preterm birth [21]. This set was obtained via extensive literature curation and imputation. Lastly, we compared significant DMRs to a gene set linked evolutionarily to mammalian pregnancy [22]. In this work Lynch et al. explore the evolution of pregnancy in placental mammals and identify 1532 gene that are uniquely expressed in the endometrium. Many of these genes were in close proximity to MER20, which regulate gene expression in response to progesterone and cAMP. These genes were broken down into gain and loss of expression in response to the stimuli.

### Pathway analysis

Pathway analysis of the genes nearest to the DMRs with *p* < 0.01 was performed using QIAGEN’s Ingenuity Pathway Analysis (www.qiagen.com/ingenuity).

### Statistical analysis

The Student’s t-test was used to evaluate significant differences between cases and controls. A two- tailed *p* < .05 was considered to indicate statistical significant difference.

## Additional files

Additional file 1: Clinical characteristics of sampled patients. (DOCX 14 kb)

Additional file 2: DMRs Associated with Preterm Birth. A tab delimitated table containing information for each DMR that was found to be associated to preterm birth with *p* < .01. The columns contain: Chromosome, DMR start location, DMR end location, with of DMR, base expression, log2FoldChange, *p* value, functional annotation and annotated nearest gene. (XLSX 55 kb)

Additional file 3: Annotation of the differentially methylated regions associated to preterm birth: CHipSeeker was used to annotate the 393 DMR (*p* < 0.01) with its corresponding genomic feature which is dependent on its genomic location. The highest percentage of DMRs is located in distal intergenic regions followed by introns. (PNG 119 kb)

Additional file 4: DMRs Associated with Gestational Age. A tab delimitated table containing information for each DMR that was found to be associated to gestational age with p < .01. The columns contain: Chromosome, DMR start location, DMR end location, with of DMR, base expression, log2FoldChange, p value, functional annotation and annotated nearest gene. (XLSX 80 kb)

## Data Availability

The dataset supporting the conclusions of this article is available in the GEO repository, under the following accession number: *GSE120458* (https://www.ncbi.nlm.nih.gov/geo/query/acc.cgi?acc=GSE120458).

## Abbreviations

BWA: Burrows-Wheeler Aligner
dbPTB: Database for Preterm Birth
DMR: Differentially methylated regions
DOHaD: Developmental Origins and Health and Disease
FDR: False discovery rate
GEO: Gene expression omnibus
IgG: Immunoglobulin G
IPA: Ingenuity Pathway Analysis
MACS: Model-based Analysis for ChIP-Seq
MeDIP-seq: Methylation dependent immunoprecipitation sequencing
NO: Nitric Oxide
