# Supplementary material for: Effect of prematurity on genome wide methylation in the placenta
Source: BMC Med Genet. 2019 Jun 28;20:116. doi: 10.1186/s12881-019-0835-6 (PMC6599230; doi:10.1186/s12881-019-0835-6)
Supplement: Supplementary file 1 — Clinical characteristics of sampled patients. (DOCX 14 kb) [file 12881_2019_835_MOESM1_ESM.docx]

| **Sample** | **Gestational Age** | **BMI** | **BW** | **Gender** | **Delivery** | **ROM** | **Drugs** | **Smoking** | **PEC** |
| --- | --- | --- | --- | --- | --- | --- | --- | --- | --- |
| Sample1 | 26 | na | 900 | M | C/S | intact | no | no | no |
| Sample2 | 37 | 54.7 | 2725 | M | Vag | at birth | no | no | no |
| Sample3 | 32 | 23 | 2205 | M | Vag | at birth | no | Yes | no |
| Sample4 | 33 | 31.85 | 2060 | F | Vag | at birth | no | no | no |
| Sample5 | 30 | 40 | 1790 | F | C/S | intact | no | no | hypertension |
| Sample6 | 34 | 21.87 | 1600 | M | C/S | intact | no | no | Yes |
| Sample7 | 25 | 37.3 | 690 | F | Vag | PROM | no | no | no |
| Sample8 | 40 | 43.1 | 3115 | F | Vag | at birth | no | no | no |
| Sample9 | 41 | 24.3 | 3260 | M | Vag | at birth | no | no | no |

**AdditionalFile 1: Clinical Characteristics of Sampled Patients.**
