# Supplementary figures and images for: Effect of prematurity on genome wide methylation in the placenta
Source: BMC Med Genet. 2019 Jun 28;20:116. doi: 10.1186/s12881-019-0835-6 (PMC6599230; doi:10.1186/s12881-019-0835-6)

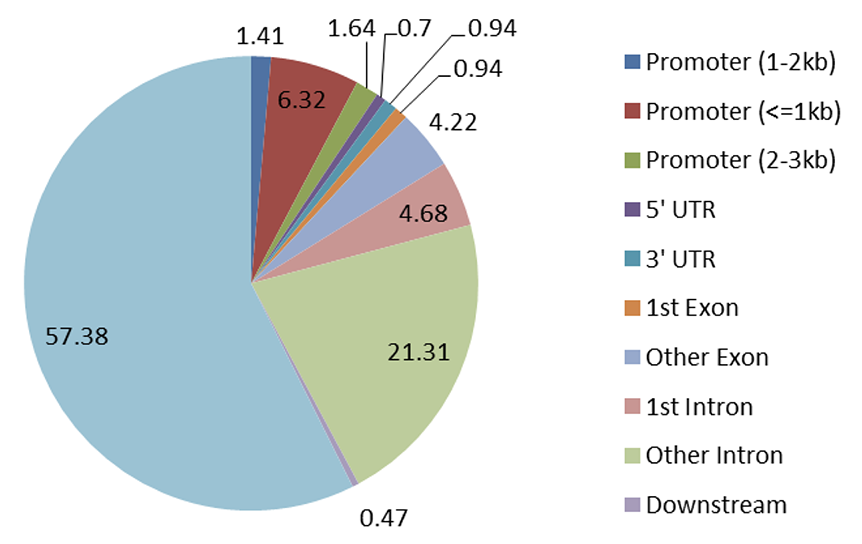

Supplement: Supplementary file 3 — Annotation of the differentially methylated regions associated to preterm birth: CHipSeeker was used to annotate the 393 DMR (p < 0.01) with its corresponding genomic feature which is dependent on its genomic location. The highest percentage of DMRs is located in distal intergenic regions followed by introns. (PNG 119 kb) [file 12881_2019_835_MOESM3_ESM.png]
